# Career aspirations among specialty residents in France: a cross-sectional gender-based comparison

**DOI:** 10.1186/s12909-021-02494-1

**Published:** 2021-01-19

**Authors:** A. Cathelain, M. Jourdain, C. Cordonnier, S. Catteau-Jonard, D. Sebbane, M. C. Copin, L. Berlingo, C. Rubod, C. Garabedian

**Affiliations:** 1 CHU Lille, Department of Obstetrics & Gynecology, Lille University Hospital, Avenue Eugène Avinée, F-59000 Lille, France; 2 INSERM, CHU Lille, U1190, Transrational diabetes research, Intensive Medicine and Resuscitation Department, F-59000 Lille, France; 3 Department of Neurology, CHU Lille, Inserm U1171, Degenerative and Vascular Cognitive Disorders, Lille University Hospital, Lille, France; 4 CHU Lille, Department Endocrine Gynecology and Reproductive Medicine, Lille University Hospital, F-59000 Lille, France; 5 CHU Lille, Department of psyciatry, F-59000 Lille, France; 6 ECEVE, UMRS 1123, Université Paris Diderot, Sorbonne Paris Cité, INSERM, Paris, France; 7 CHU Lille, Pathology Institute, Lille University Hospital Center, F-59000 Lille, France; 8 Maternity, Pitié Salpêtrière Hospital, Assistance Publique des Hôpitaux de Paris, Sorbonne University, Paris, France

**Keywords:** Gender, University career, Internship, Women, Medicine

## Abstract

**Background:**

Most studies evaluating career aspirations among gender are performed in Anglo-Saxon countries. Two recent French studies looked at the career choice of residents in obstetrics & gynecology. It seemed useful to us to broaden this questioning to other specialties, by proposing a study to all residents in the same Faculty. The objective of our study was to describe residents’ career aspirations and possible barriers according to gender.

**Methods:**

Declarative cross-sectional survey, using questionnaires sent by email to the specialty residents of the Faculty of Medicine of Lille (France). An analysis by specialty group (i.e., medicine, surgery, obstetrics & gynecology, and anesthesia & resuscitation) and a comparison of the results according to gender were performed.

**Results:**

Of the 1384 specialty residents currently in training, 462 answered the questionnaire (33.38%), among whom 289 women and 173 men (average age = 27.08 ± 0.091 years). Seventeen women (5.9%) were currently considering a university hospital career versus 37 men (21.4%) (*p* = 0.001).

Gender analysis made it possible to identify obstacles to engaging in a university career: lacking a female model, more frequent doubting the ability to undertake this type of career among women (61.6%) than men (35.3%) (*p* < 0.001), and gender discrimination felt in the workplace for 51.6% of women (versus 7.5% of men, *p* < 0.001). Subgroup analysis showed specificities related to each specialty.

**Conclusions:**

Few residents plan to embark upon a university hospital career, let alone female residents. There are considerations specific to each specialty and marked gender differences regarding career aspirations. Many features have been identified as obstacles to access to university hospital positions for women. It is important to develop strategies to remove these barriers and enable women to pursue such university careers.

**Trial registration:**

Not applicable (no intervention).

**Supplementary Information:**

The online version contains supplementary material available at 10.1186/s12909-021-02494-1.

## Background

The themes of equality between men and women, career choices, and personal–professional balance are burning issues, with many studies published and reported by the media. Several recent studies have addressed questions of personal–professional balance: the balance between professional as well as personal constraints and satisfactions, discriminatory barriers to desirable positions, and career aspirations [1–5].

These studies provide a state-of-play of hospital functioning and describe the place of women in positions of responsibility within the medical community. The study by Rosso et al. described the presence of a glass ceiling and detailed the obstacles to the progression of women in the university hospital environment in France [1]. The recent study by Carr et al. described the same phenomenon of a glass ceiling in a cohort of faculty from the 1995 National Faculty Survey in the United States [6].

Two recent additional studies looked at the career choice of residents in obstetrics & gynecology [3, 4]. They showed that many barriers limited women’s access to university hospital positions.

In a column published in a major French daily newspaper (“*Le Monde*”), a group of doctors called on the government to evaluate thoroughly the place of women in university hospital careers in France to promote their access to health management positions [7].

Indeed, according to the meta-analysis by Edmunds et al. [8], women show less interest in research than in education, suffer gender-based discrimination, and lack mentors. However, most of the studies cited in this meta-analysis have been carried out in Anglo-Saxon countries. Thus, the conclusions do not necessarily correspond to the reasons that can be identified in France. Faced with this observation, two consecutive studies were carried out in 2017 and 2018 among residents in obstetrics & gynecology, a specialty mainly including women (respectively 85 and 86% of female residents in Paris in 2016–2017 and in Lille in 2017–2018, respectively). In these studies [3, 4], there was less attraction for university hospital positions among women due to clearly identified obstacles. These were the lack of mentor and female models which would make it possible to create vocations, difficulties in time management linked to clinical, research, and teaching activities together, which characterizes the French university hospital model, and a lower interest in research on women’s part. As Scanlan et al. pointed out, many studies have been done on a single specialty or on small numbers of students, but few on a group of students from several specialties [9].

With these findings, it seemed useful to us to broaden this questioning to other specialties than obstetrics & gynecology, by proposing a study available to all Lille residents. Thus, the objective of our study was to assess career aspirations and obstacles within all residents of the Faculty of Medicine of Lille, according to gender and specialty profile (medical, surgical, anesthesia & resuscitation, and obstetrics & gynecology).

## Methods

This was a declarative cross-sectional survey of all specialty residents in the Faculty of Medicine in Lille (France). This consisted of answering a questionnaire made up of 40 items, sent by email, via Google Forms. The questionnaire was titled “Choosing a University Career Among Lille’s Specialty Residents.” This questionnaire was anonymous. It was mandatory to respond to every question, therefore, we do not have any missing data. The selected population responded to it voluntarily. It was posted via Facebook using a permanent link to the group of the Association of Residents in Practice of Lille Hospitals (AIEHL). It was also distributed by email by the respective associations of residents of each specialty between January and June 2019.

In France, the training period of a resident lasts 4 to 5 years depending on the specialty. The study population included all residents undergoing training registered at the Faculty of Medicine of Lille (France), from the first year to the last inclusive. General medicine residents were not included in this study because they are rarely concerned with university hospital careers. General medicine has been recognized as a specialty only since 2004 in France. However, general practitioners still practice a lot in private. In 2018, less than 20% of general practitioners were employed in hospitals versus 40% of specialists [10].

The current number of Lille residents was 1384 according to data from the Regional Health Agency (ARS).

The questionnaire was the one developed by Berlingo et al. [3], and also used for the study by Cathelain et al. [4] on residents in obstetrics & gynecology. This questionnaire collected different types of data. Sociodemographic characteristics were collected (in particular age and biological sex) as well as career aspirations (private sector, university hospital, hospital) and the means to achieve them. The idea was to identify if they fulfilled or wished to fulfill the prerequisites to be a university hospital doctor. We then sought to identify their priorities in life, as well as the areas they deemed most important for their future development. We asked them to rank the importance of each item from 1 to 5 (1 being the least important and 5 the most important). Survey is disponible as Appendix 1.

Finally, we asked them about the possible obstacles to accessing a university career, the obstacles being identified thanks to a literature review, especially the review published by Edmunds et al. [8], such as the presence of a mentor or an academic model, support in their research projects, resentment of gender discrimination, doubts about their ability to pursue a university career, and finally if their career choice would have been different if they had been of the opposite sex.

### Statistical analyses

A univariate descriptive analysis of the data collected was carried out, then supplemented by an analysis by specialty group, divided into four subgroups: medical specialties, surgical specialties, obstetrics & gynecology, and anesthesia & resuscitation. For the numerical and ordinal variables, we used the Student t test, and for the categorical variables, we used the chi-square test. All analyses were performed using SPSS Statistics version 25 software. The article was checked against all the 22 relevant elements of the STROBE checklist.

### Ethics

The processing of personal data was carried out in accordance with the European regulations in force relating to data protection. They appear in the treatment register under the reference: N * DEC19–486. Institutional Review Board approval was not required according to the national regulation in France.

## Results

Among the 1384 residents, 462 of them (33.38%) responded to the questionnaire, divided into 289 women (62.6%) and 173 men (37.4%). The number of medical specialty residents was 293 (63.4%) with a women/men (W/M) ratio of 1.99; 47 for surgical specialty residents (10.2%) with a W/M ratio of 0.52, 67 in obstetrics & gynecology (14.5%) with a W/M ratio of 5.7, and finally, 55 in anesthesia & resuscitation (11.9%) with a W/M ratio of 0.62. No forensic, nuclear, public health, neurosurgery, ophthalmology, plastic, cardiac, and thoracic surgery residents responded to the questionnaire (*n* = 112). The details of the numbers and the response rate according to each specialty can be found in Table S1.

Table 1 presents the sociodemographic characteristics according to the type of resident interviewed and according to the specialty subgroup. We did not find any significant difference concerning age, residency semester, marital and family status, and between men and women.

**Table 1:** Social and demographic data, and career aspirations, analysis by gender

	All specialties	medical specialties			surgical specialties			Gynecology-Obstetrics			Anesthesia & resuscitation		
	total *n* = 462	men *n* = 98	women *n* = 195	*p* value	men *n* = 31	women *n* = 16	*p* value	men *n* = 10	women *n* = 57	*p* value	men *n* = 34	women *n* = 21	*p* value
Age: Mean (DS)	27,08 (0,091)	26,82 (2,04)	26,99 (1,99)	.486	27,26 (1,93)	27,38 (1,74)	.840	27,20 (1,47)	27,33 (1,97)	.840	27,56 (1,94)	27,24 (1,75)	.540
semester of residency [1–10]: Median [25–75]	5 [3;7]	5 [3;7]	5 [3;7]	.933	5 [3;8]	6,5 [5;7]	.342	4,5 [2;6]	6 [2;8]	.361	5 [3;8]	7 [5;9]	.531
marital status				.431			.931			.634			.368
single	134 (29,0%)	34 (34,7%)	54 (27,7%)		9 (29,0%)	4 (25,0%)		4 (40,0%)	16 (28,1%)		6 (17,6%)	7 (33,3%)	
attached	272 (58,9%)	54 (55,1%)	122 (62,6%)		19 (61,3%)	10 (62,5%)		4 (40,0%)	32 (56,1%)		20 (58,8%)	11 (52,4%)	
married	56 (12,1%)	10 (10,2%)	19 (9,7%)		3 (9,7%)	2 (12,5%)		2 (20,0%)	9 (15,8%)		8 (23,5%)	21 (38,2%)	
number of children				.669			.945			.052			.425
0	425 (92,0%)	10 (10,2%)	12 (6,2%)		1 (3,2%)	0 (0,0%)		0 (0,0%)	1 (1,8%)		4 (11,8%)	2 (9,5%)	
1	24 (5,2%)	4 (4,1%)	10 (5,1%)		1 (3,2%)	1 (6,3%)		2 (20,0%)	1 (1,8%)		2 (5,9%)	1 (6,3%)	
2	9 (1,9%)	48 (49,0%)	96 (49,2%)		15 (48,4%)	8 (50,0%)		5 (50,0%)	31 (54,4%)		17 (50,0%)	10 (47,6%)	
3	1 (0,2%)	26 (26,5%)	61 (31,3%)		12 (38,7%)	6 (37,5%)		2 (20,0%)	23 (40,4%)		5 (14,7%)	7 (33,3%)	
> 3	3 (0,6%)	10 (10,2%)	16 (8,2%)		2 (6,5%)	1 (6,3%)		1 (10,0%)	1 (1,8%)		6 (17,6%)	1 (4,6%)	
academic partner *				.218			.492			.271			.444
no	264/328 (80,5%)	56/64 (87,5%)	109/141(77,3%)		19/22 (86,4%)	9 (75,0%)		4/6 (66,7%)	31/41(75,6%)		23/28 (82,1%)	13/14 (92,9%)	
doesn’t know	15/328 (4,6%)	1/64 (1,6%)	6/141 (4,3%)		1/22 (4,5%)	2 (16,7%)		1/6 (16,7%)	1/41 (2,4%)		2/28 (7,1%)	1/14 (7,1%)	
yes	49/328 (14,9%)	7/64 (10,9%)	26/141 (18,4%)		2 /22(9,1%)	1 (8,3%)		1/6 (16,7%)	9/41 (22,0%)		3/28 (10,7%)	0/14 (0,0%)	
partner support *				.433			.904			.257			.472
no	27/328 (8,2%)	8/64 (12,5%)	10/141(7,1%)		2/22 (9,1%)	1/12 (8,3%)		1/6 (16,7%)	1/41(2,4%)		2/28 (7,1%)	2/14 (14,3%)	
doesn’t know	17/328 (5,2%)	4/64 (6,3%)	8/141 (5,7%)		1/22 (4,5%)	1/12 (8,3%)		0/6 (0,0%)	1/41 (2,4%)		2/28 (7,1%)	2/14 (14,3%)	
yes	284/328 (86,6%)	52/64 (81,3%)	123/141 (87,2%)		19/22 (86,4%)	10/12 (83,3%)		5/6 (83,3%)	39/41 (95,1%)		24/28 (85,7%)	12/14 (85,7%)	
academic father	62 (13,4%)	13 (13,3%)	27 (13,8%)	.891	6 (19,4%)	1 (6,3%)	.396	1 (10,0%)	3 (5,3%)	.560	9 (26,5%)	2 (9,5%)	.174
academic mother	80 (17,3%)	14 (14,3%)	44 (22,6%)	.093	5 (16,1%)	4 (25,0%)	.466	3 (30,0%)	3 (5,3%)	.120	5 (14,7%)	2 (9,5%)	.696

*only if attached or married

Concerning career aspirations according to sex and type of specialty (Table 2), men wanted more than women to pursue research (35.3% versus 19.4%, *p* = 0.001) and teaching (61.3% versus 32.5%, *p* < 0.001). Seventeen women (5.9%) were currently considering a university hospital career compared with 37 men (21.4%), (*p* = 0.001). In obstetrics & gynecology, 70% of men wanted to do a Master of Research against 15.8% of women (*p* = 0.001). In medical specialties, women published fewer articles than men (38.5% versus 55.1%, *p* = 0.035) and wished less often to become Assistant Head of the University Hospitals (CCA-HU) (29.2%) than men (44.9%), *p* = 0.049. Finally, in surgical specialties, no woman had a vocation for a university hospital career, compared with 9.7% of men, *p* = 0.039.

**Table 2:** Career aspirations, analysis by gender

	All specialties	Medical specialties			Surgical specialties			Gynecology-Obstetrics			Anesthesia & resuscitation		
	total *n* = 462	men *n* = 98	women *n* = 195	*p* value	men *n* = 31	women *n* = 16	*p* value	men *n* = 10	women *n* = 57	*p* value	men *n* = 34	women *n* = 21	*p* value
Master of science				.227			.103			.001			.212
unintended	267 (57,8%)	46 (46,9%)	114 (58,5%)		19 (61,3%)	12 (75,0%)		2 (20,0%)	32 (56,1%)		23 (67,6%)	19 (90,5%)	
don’t know	36 (7,8%)	5 (5,1%)	15 (7,7%)		4 (12,9%)	1 (6,3%)		0 (0,0%)	8 (14,0%)		3 (8,8%)	0 (0,0%)	
wished but not expected	41 (8,9%)	13 (13,3%)	19 (9,7%)		2 (6,5%)	1 (6,3%)		3 (30,0%)	1 (1,8%)		2 (5,9%)	0 (0,0%)	
desired and planned	62 (13,4%)	16 (16,3%)	23 (11,8%)		6 (19,4%)	0 (0,0%)		4 (40,0%)	8 (14,0%)		3 (8,8%)	2 (9,5%)	
already achieved	56 (12,1%)	18 (18,4%)	24 (12,3%)		0 (0,0%)	2 (12,5%)		1 (10,0%)	8 (14,0%)		3 (8,8%)	0 (0,0%)	
PhD in science				.151			.240			.004			.084
unintended	326 (79,6%)	58 (59,2%)	140 (71,8%)		23 (74,2%)	15 (93,8%)		4 (40,0%)	40 (70,2%)		25 (73,5%)	21 (100%)	
don’t know	69 (14,9%)	17 (17,3%)	24 (12,3%)		5 (16,1%)	1 (6,3%)		2 (20,0%)	14 (24,6%)		6 (17,6%)	0 (0,0%)	
wished but not expected	16 (3,5%)	7 (7,1%)	7 (3,6%)		0 (0,0%)	0 (0,0%)		0 (0,0%)	1 (1,8%)		1 (2,9%)	0 (0,0%)	
desired and planned	48 (10,4%)	16 (16,3%)	22 (11,3%)		3 (9,7%)	0 (0,0%)		3 (30,0%)	2 (3,5%)		2 (5,9%)	0 (0,0%)	
already achieved	3 (0,6%)	0 (0%)	2 (1,0%)		0 (0,0%)	0 (0,0%)		1 (10,0%)	0 (0,0%)		0 (0,0%)	0 (0,0%)	
Publication article				.035			.412			.340			.378
none	257 (55,6%)	44 (44,9%)	120 (61,5%)		14 (45,2%)	7 (43,8%)		6 (60,0%)	28 (49,1%)		21 (61,8)	17 (44,7%)	
in progress	132 (28,6%)	38 (38,8%)	46 (23,6%)		11 (35,5%)	8 (50,0%)		1 (10,0%)	19 (33,3%)		6 (17,6%)	3 (14,3%)	
1	47 (10,2%)	10 (10,2%)	17 (8,7%)		6 (19,4%)	1 (6,3%)		2 (20,0%)	4 (7,0%)		6 (17,6%)	1 (4,8%)	
> 1	26 (5,6%)	6 (6,1%)	12 (6,2%)		0 (0,0%)	0 (0,0%)		1 (10,0%)	6 (10,5%)		1 (2,9%)	0 (0,0%)	
Post-residency				.049			.075			.001			.661
PHC-attach	24 (5,2%)	6 (6,1%)	10 (5,1%)		0 (0,0%)	1 (6,3%)		0 (0,0%)	0 (0,0%)		4 (11,8%)	3 (14,3%)	
assistant	199 (43,1%)	33 (33,7%)	91 (46,7%)		9 (29,0%)	9 (56,3%)		2 (20,0%)	31 (54,4%)		13 (38,2%)	11 (52,4%)	
doesn’t know	79 (17,1%)	15 (15,3%)	37 (19,0%)		3 (9,7%)	2 (12,5%)		0 (0,0%)	13 (22,8%)		6 (17,6%)	3 (14,3%)	
fellowship	160 (34,6%)	44 (44,9%)	57 (29,2%)		19 (61,3%)	4 (25,0%)		8 (80,0%)	13 (22,8%)		11 (32,4%)	4 (19,0%)	
Wants to do research	117 (25,3%)	37 (37,8%)	49 (25,1%)	.067	6 (19,4%)	2 (12,5%)	.608	5 (50,0%)	4 (7,0%)	.001	13 (38,2%)	1 (4,8%)	.012
Wants to teach	200 (43,3%)	57 (58,2%)	70 (35,9%)	.001	20 (64,5%)	2 (12,5%)	.002	9 (90,0%)	19 (33,3%)	.003	20 (58,8%)	3 (14,3%)	.002
Career envisioned				<.001			.039			<.001			.810
private practice	77 (16,7%)	17 (17,3%)	34 (17,4%)		12 (38,7%)	2 (12,5%)		0 (0,0%)	6 (10,5%)		4 (11,8%)	2 (9,5%)	
hospital staff physician	220 (47,6%)	39 (39,8%)	96 (49,2%)		10 (32,3%)	12 (75,0%)		5 (50,0%)	23 (40,4%)		20 (58,8%)	15 (71,4%)	
academic medicien	54 (11,7%)	26 (26,5%)	14 (7,2%)		3 (9,7%)	0 (0,0%)		5 (50,0%)	2 (3,5%)		3 (8,8%)	1 (4,8%)	
doesn’t know	111 (24,0%)	16 (16,3%)	51 (26,2%)		6 (19,4%)	2 (12,5%)		0 (0,0%)	26 (45,6%)		7 (20,6%)	3 (14,3%)	

Table 3 presents residents’ priorities for personal development, according to sex and type of specialty. Family life and freedom of choice in working hours were more important for women than for men (*p* = 0.001), while knowledge transfer was more important for men than for women (*p* = 0.012). Interest in intellectual stimulation was higher among men in medical specialties (*p* = 0.006). Men in surgical specialties declared social recognition as a higher priority (*p* = 0.014). There was no gender priority difference among residents in anesthesia & resuscitation. Possible barriers to a university career, according to gender and type of specialty, are detailed in Table 4. Male residents received more advice for their future career from seniors than female residents did (52.6% versus 41.9%, *p* = 0.016). They had less doubt than women about their ability to pursue a university career (35.3% versus 61.6%, *p* < 0.001). 56.1% of female residents had already experienced discrimination related to their gender, compared with 7.5% of male residents (*p* < 0.001). In surgery, among seniors who stand for university models for residents, 95.5% are the same sex as male residents, and 28.6% are the same sex as female residents (*p* < 0.001). In medical specialties, 39% of women thought that their career choice would have been different if they had been of the opposite sex, compared with 17.3% of men (*p* < 0.001). Finally, in anesthesia & resuscitation, 52.9% of men thought it was possible to reconcile clinical, research, and teaching activities against 23.8% of women (*p* = 0.033). Table S2 presents the results for all specialties combined.

**Table 3:** Priorities, analysis by gender Question asked: ‘For the following elements, please rank from 1 (least important) to 5 (most important) their importance for your future self-fulfilment?’ Mean (DS)

	All specialties	Medical specialties			Surgical specialties			Gynecology-Obstetrics			Anesthesia & resuscitation		
	total *n* = 462	men *n* = 98	women *n* = 195	*p* value	men *n* = 31	women *n* = 16	*p* value	men *n* = 10	women *n* = 57	*p* value	men *n* = 34	women *n* = 21	*p* value
Family Life	4,50 (0,04)	4,24 (0,94)	4,61 (0,73)	<.001	4,65 (0,61)	4,56 (0,51)	.644	4,30 (0,82)	4,54 (0,80)	.381	4,35 (0,98)	4,76 (0,70)	.102
Leisure	4,18 (0,04)	4,40 (0,62)	4,15 (0,76)	.006	4,03 (0,79)	3,81 (0,91)	.397	4,10 (0,56)	4,18 (0,71)	.715	4,12 (0,91)	4,00 (0,78)	.626
Freedom of timetables	4,09 (0,04)	4,08 (0,78)	4,06 (0,79)	.838	4,35 (0,71)	3,94 (0,57)	.048	4,20 (0,42)	4,00 (0,56)	.292	4,18 (0,83)	4,14 (0,65)	.876
Financial Compensation	4,06 (0,04)	4,02 (0,96)	4,07 (0,95)	.665	4,10 (0,91)	3,81 (1,05)	.339	4,00 (1,05)	4,04 (0,77)	.901	4,18 (0,87)	4,29 (0,72)	.631
Social Recognition	3,76 (0,04)	3,44 (1,14)	3,98 (0,93)	<.001	3,55 (1,15)	3,50 (1,03)	.888	3,30 (1,16)	3,82 (0,93)	.117	3,68 (0,91)	3,95 (0,59)	.223
Interest of the professional activity	3,63 (0,04)	3,48 (1,06)	3,54 (0,87)	.584	4,23 (0,92)	3,75 (0,93)	.101	3,60 (1,07)	3,56 (0,82)	.897	3,94 (1,01)	3,90 (0,99)	.897
Intellectual stimulation	3,39 (0,05)	3,54 (1,18)	3,29 (1,01)	.057	3,65 (0,91)	2,69 (1,13)	.003	4,30 (0,82)	3,53 (0,98)	.022	3,26 (1,16)	3,19 (0,60)	.788
Transmission of knowledge	3,03 (0,05)	3,04 (1,17)	2,94 (1,08)	.459	3,61 (1,05)	2,88 (0,62)	.014	2,90 (1,19)	3,40 (1,03)	.169	2,65 (1,20)	2,86 (0,964)	.502

**Table 4:** Potential obstacles to an academic career, in the total population and among those with academic aspirations, analysis by gender

	All specialties	Medical specialties			Surgical specialties			Gynecology-Obstetrics			Anesthesia & resuscitation		
	total *n* = 462	men *n* = 98	women *n* = 195	*p* value	men *n* = 31	women *n* = 16	*p* value	men *n* = 10	women *n* = 57	*p* value	men *n* = 34	women *n* = 21	*p* value
Do you get advice from anacademic physician about your future career? Yes (n,%)	212 (45,9%)	57 (58,2%)	87 (44,6%)	.029	11 (35,5%)	7 (43,8%)	.581	8 (80,0%)	21 (36,8%)	.011	19 (55,9%)	15 (71,4%)	.249
Do you feel you are supported when you work on a research project? Yes * (n,%)	145/229 (63,3%)	39/51 (76,4%)	58/100 (58,0%)	.080	10/20 (50,0%)	6/8 (75,0%)	.325	5/5 (100,0%)	17/21 (62,9%)	.368	6/15 (40,0%)	4/9 (44,4%)	.973
Do you think that it’s possible to reconcile (interms of time) research activity, teaching and clinical practice? Yes (n,%)	213 (46,1%)	46 (46,9%)	83 (42,6%)	.477	14 (45,2%)	8 (50,0%)	.753	9 (90,0%)	30 (52,6%)	.027	18 (52,9%)	5 (23,8%)	.033
Among the academic doctors on staff who you know, would you say that some are models for you? Yes (n,%)	297 (64,3%)	69 (70,4%)	120 (61,5%)	.134	22 (71,0%)	7 (43,8%)	.069	10 (100%)	38 (66,7%)	.031	18 (52,9%)	13 (61,9%)	.515
If yes, are they mainly the same sex as you? Yes (n,%)	150/297 (50,5%)	43/69 (62,3%)	44/120 (36,7%)	.001	21/22 (95,5%)	2/7 (28,6%)	<.001	7/10 (70,0%)	18/38 (47,4%)	.202	12/18 (66,7%)	3/13 (23,1%)	.017
Have you already experienced discrimination or prejudice due to your gender? Yes (n,%)	175 (37,9%)	6 (6,1%)	111 (56,9%)	<.001	3 (9,7%)	13 (81,3%)	<.001	2 (20,0%)	23 (40,4%)	.209	2 (5,9%)	15 (71,4%)	<.001
Do you have doubts about your ability to pursue or succeed at an academic career? Yes (n,%)	239 (51,7%)	40 (40,8%)	129 (66,2%)	< .001	10 (32,3%)	6 (37,5%)	.866	3 (30,0%)	34 (59,6%)	.219	8 (23,5%)	9 (42,9%)	.308
Do you think that your career plans would have been different if you were a member of the opposite sex? Yes (n,%)	134 (29,0%)	17 (17,3%)	76 (39,0%)	<.001	11 (35,5%)	3 (18,8%)	.423	2 (20,0%)	16 (28,1%)	.392	4 (11,8%)	5 (23,8%)	.152

*only for residents with a research project

## Discussion

In recent years, we have witnessed a feminization of the medical profession, but the highest positions in hierarchies within universities and hospitals remain predominantly occupied by men. In our study, we show that women plan less than men to pursue a university career, and this difference is even more marked in certain specialties such as surgery. Whatever the specialty, women favor a career in a hospital environment. Presumably, this choice is likely linked to the benefits of the wage system, which provides job security and pay, especially among women of childbearing age. In fact, according to the study by Pyatigorskaya et al. concerning residents in radiology, maternity remains a source of inequality in France and implies that a greater number of women are ready to take up salaried positions to obtain secure employment, even if their remuneration may be lower [11]. 74% of women declared that motherhood could influence their career choice, and were less interested in unstable positions exclusively in private practice (45% of men and 33% of women, *p* = 0.05) [11]. In the study by Cochran et al., almost half of female surgeons agreed or strongly agreed that having children would be a barrier to their careers, compared with only 5% of their male colleagues [12].

For some women, the choice of career type had not yet been decided: we note that more women answered: “do not know” to the question on the career envisaged, compared with men. The fact of not making a choice, or of hesitating, is something very present in the responses of women: to the question “do you doubt your ability to achieve a university career?” two-thirds of women answered “yes.” In 2020, Manne-Goehler et al. studied the relationship between self-esteem, gender, and career outcomes. Women had lower self-esteem than men [13].

This doubt may also be related to the fact that women receive less advice about their future careers from university doctors than men do. It may also be related to the fact that when women have an academic model, this model is generally not of the same sex as them, which makes it more difficult to project themselves into an ideal of career. In surgery, for example, no female resident wished to pursue a university hospital career, and at the same time, we note that almost all of the men declared having a model of the same sex, compared with less than a third of females. Numerous studies have shown the mentor’s role in choosing careers [14–21], and more particularly for women in surgery, with the recent study by Bettis et al. [22]. Concerning the problem of discrimination running from the start of medical studies, it is already well known and described [23–26]. In our study, we find major discrimination rates in surgery and anesthesia & resuscitation, which are still predominantly male specialties. We think that this significant rate of discrimination is linked to the place of practice of these specialties: the operating room. Even if mentalities have changed a lot, the operating room remains a place where a special atmosphere prevails. Relations are sometimes tense between medical and paramedical teams, in relation to the level of stress of the doctors, the arduousness of the work, and the organizational constraints of each. The operating room can be close to a sort of in-camera for the teams, a confined place where overflows, such as verbal abuse or harassment, can easily be trivialized.

The last point specific to university careers is that the triple activity of research, teaching, and clinical practice seems difficult to manage for women, specifically in anesthesia & resuscitation and obstetrics & gynecology. In addition, women seem to be less interested in research than in teaching, and overall less interested in transmitting knowledge than men.

Regarding priorities in life, overall, for all residents and even more markedly for women, family life remains the most important for their future development. Interest in professional activity and intellectual stimulation are also important priorities. On the other hand, social recognition and salary are not ranked at the top of the priorities, except for male surgeons who classify them as relatively important. It is, therefore, important to take into consideration the challenges linked to the balance between professional career and personal life, and the priorities of each, according to the specific characteristics of specialties and gender.

Studies that seek to identify the obstacles to a university career for women are increasing [3, 4, 8, 11, 27]. In 2016, Bates et al. published some ideas to reduce barriers and for gender parity [28]. In February 2019, The Lancet published a thematic issue (#LancetWomen) on the place of women in science, medicine, and world health to highlight and promote research work in favor of gender equality [29]. It was clearly established that women are underrepresented in leadership, decision-making, and research [30]. This special issue highlighted the importance of gender parity in medical and scientific teams. For example, the work by Sugimoto et al. showed us the importance of more diverse and inclusive teams, particularly with regard to the declaration of the sex of participants and the inclusion of women in clinical studies [31]. Regarding the existing discrimination between men and women, the work by Witteman et al. raised the problem of access to research grants, which are preferentially awarded to men [32]. The editorial in this special issue concluded that it is the responsibility of everyone—researchers, clinicians, institutional leaders, and even medical journals—to promote gender equality.

Faced with these findings of inequality at different levels in science and medicine, it seems important to put in place concrete strategies from the start of medical studies. For example, in the United States, following surveys carried out by the Association of American Medical Colleges (AAMC), different strategies have been implemented: the “Women in Medicine and Health Science” (WIMHS) program started in 2000 in California and the “Group on Women in Medicine and Science” (GWIMS) was created in the United States in 2009 to attract women to university medicine. These programs have had a positive influence on the recruitment and job satisfaction of women, with a constant increase in the number of women teachers and in service management [33, 34]. In France, there is currently no strategy for the specific supervision of female interns. However, since November 2017, a reform in medical studies provides for supervision provided by a tutor, from the start of residency. The training contract and the portfolio allow the individualization of the training path to meet the student’s professional project, as well as the personalization of their follow-up [35]. We hope that women can benefit from this reform thanks to personalized support to help them embark on a university career if they wish. It seems important to inform women early in their studies about possible careers.

Our study has strengths but also certain limitations. Concerning the response rate, more than a third of Lille hospital residents responded to our survey. This is around that expected for a study using online survey administration; however, this creates the potential for response biases to influence study findings. However, there are disparities according to the specialties: more than 90% of respondents in obstetrics & gynecology, and less than 30% of respondents in surgery (Table S1). Concerning the sex ratio of the respondents, it is difficult to know if it is representative because the ARS does not have data on the gender or the sex of specialties’ interns. It would have been interesting to strengthen our findings. In total, more women than men responded to our study, but we cannot know whether this is solely related to the feminization of the profession, or to the fact that women are more interested in this subject. This would then imply a response bias.

The originality of our study is to have interviewed the young generation of future doctors and/or surgeons within the same Faculty of Medicine, without being limited to a single specialty.

Finally, those results could be generalized to other centers. Indeed, the Lille University Hospital is one of the most important university centers in France with an large population of residents. The population studied is that of residents with a training program similar to other European countries. The strength of this study is also to evaluate the choice of career whatever the specialty, medical or surgical. Apart from this specialization in France, the curriculum is the same in most countries. Finally, the generalizability of the results of this study is also in line with previous studies undertaken in Anglo-Saxon countries, which have given a worldwide character to this topic.

## Conclusions

Female medical residents are less likely than their male counterparts to pursue a university career, and this difference is even more marked in some specialties. The main obstacles identified are the same in all specialties. Women doubt their ability to pursue a university hospital career, their legitimacy to access such a position, and self-censor. Most of today’s hospital doctors are men: women in the process of being trained lack a mentor and female models that would encourage vocations. The triple clinical/research/teaching activity that characterizes the French university hospital model seems to be a hindrance due to the time management which seems difficult between the three aspects, and a lesser interest in research and teaching in women. As more and more women work in the medical profession, it is important to develop strategies to encourage and support those who wish to pursue a university hospital career. It is also important to think about the quality of life of future doctors, male or female, whose work–family balance is the main concern.

## Supplementary Information

**Additional file 1.** Survey: Career aspirations among specialty residents (in English).

**Additional file 2.** **Table S1** : Responses by specialty.

**Additional file 3.** **Table S2** : Results for all specialties.

## Data Availability

The datasets used and/or analysed during the current study available from the corresponding author on reasonable request.
