# Supplementary material for: Career aspirations among specialty residents in France: a cross-sectional gender-based comparison
Source: BMC Med Educ. 2021 Jan 19;21:63. doi: 10.1186/s12909-021-02494-1 (PMC7816478; doi:10.1186/s12909-021-02494-1)
Supplement: Supplementary file 1 — Additional file 1. Survey: Career aspirations among specialty residents (in English). [file 12909_2021_2494_MOESM1_ESM.docx]

**Survey : Career aspirations among specialty residents (in English)**

**- Are you a man or a woman?** M/W

**- How old are you?**

**- What is your specialty?**

**- What semester are you currently in?**

**- What is your family situation?** single / in couple / married

**- If as a couple or married :**

**- What is your spouse's occupation?**

**- Does your spouse have academic aspirations?** Yes / No / Don't know

**- Is your spouse a support in your career choice?** Yes / No / Don't know

**- Do you have children?** 0 / 1 / 2 / 3 / >3

**- How many children do you wish to have (in total) in the future?** 0 / 1 / 2 / 3 / >3

**- Does your father have a job related to research or teaching (in medicine or other fields)?** Yes / No

**- Does your mother have a job related to research or teaching (in medicine or other fields)?** Yes / No

**- Would you like to make a « master 2 »?**

not wished / wished but not planned / wished and planned / already realized / don't know

**- Would you like to do a science thesis?**

not wished / wished but not planned / wished and planned / already realized / don't know

**- Have you ever published scientific articles?**

none / in progress / 1 / >1

**- Would you like to give internship lectures?**

not wished / wished but not planned / wished and planned / already done / don't know

**- Do you wish to carry out a mobility abroad (semester, master, thesis or other research project)?**

not wished / wished but not planned / wished and planned / already done / don't know

**- For the immediate post-internship, do you have a position planned?** yes / no

**- In the immediate post-internship period, what type of position would you like or plan to have?** PHC-attaché / assistant / CCA

**- Would you like to have a research activity in the future?** Yes / No / don't know

**- Would you like to have a teaching activity in the future?** Yes / No / don't know

**- Are you considering a career in the future?**

university hospital (MCU-PH, PU-PH) / in hospital / in the private sector

**- Have you ever received advice from a university hospital physician (MCU-PH or PU-PH) regarding the direction of your future career?** Yes / No / don't know

**- Do you feel supported when conducting a research project?** Yes / No / no research project at this time

**- Do you think it is possible to reconcile (in terms of time) research activity, teaching activity and clinical activity?** Yes / No

**- Among the university hospital doctors you knew, would you say that some are role models for you?** Yes / No

**- If so, are they more of the same sex as you?** Yes / No

**- Have you ever experienced discrimination or prejudice due to your gender?** Yes / No / don't know

**- Do you have any doubts about your ability to pursue an academic career?** Yes / No / don't know

**- Do you think your career plans would have been different if you had been of the opposite sex?** Yes / No / don't know

**With respect to the following items, can you rate on a scale of 1 to 5 (1 being least important, 5 being most important) how important they are to your future development?**

**- Interest in the work activity** 1 2 3 4 5

**- Family life** 1 2 3 4 5

**- Recreation** 1 2 3 4 5

**- Intellectual stimulation** 1 2 3 4 5

**- Social Recognition** 1 2 3 4 5

**- Freedom of scheduling** 1 2 3 4 5

**- Financial compensation** 1 2 3 4 5

**- Knowledge transfer** 1 2 3 4 5
