# Supplementary material for: Career aspirations among specialty residents in France: a cross-sectional gender-based comparison
Source: BMC Med Educ. 2021 Jan 19;21:63. doi: 10.1186/s12909-021-02494-1 (PMC7816478; doi:10.1186/s12909-021-02494-1)
Supplement: Supplementary file 2 — Additional file 2. Table S1 : Responses by specialty. [file 12909_2021_2494_MOESM2_ESM.docx]

| Table S1 : Responses by specialty | | | | | |
| --- | --- | --- | --- | --- | --- |
| Specialties | total sample n=462 | men n= 173 (37,4%) | women n= 289 (62,6%) | total number of resident (for specialties  respondents to the questionnaire) n = 1272 | response rate  by specialty |
| Medical Specialties | | | | | |
| Allergology | 2 (0,43%) | 1 (50%) | 1 (50%) | 2 (0,16%) | 100,00% |
| Anatomy and cytology pathology | 11 (2,38%) | 3 (27,3%) | 8 (72,7%) | 15 (1,18%) | 73,33% |
| Cardiology | 21 (4,55%) | 11 (52,4%) | 10 (47,6%) | 52 (4,09%) | 40,38% |
| Dermatology | 12 (2,60%) | 3 (25%) | 9 (75%) | 26 (2,05%) | 46,15% |
| Endocrinology | 19 (4,11%) | 6 (31,6%) | 13 (68,4%) | 31 (2,44%) | 61,29% |
| Gastroenterology and Hepatology | 8 (1,73%) | 2 (25%) | 6 (75%) | 33 (2,60%) | 24,24% |
| Medical Genetics | 1 (0,22%) | 0 (0%) | 1 (100%) | 4 (0,31%) | 25,00% |
| Geriatrics | 6 (1,30%) | 1 (16,7%) | 5 (83,3%) | 25 (1,97%) | 24,00% |
| Medical Gynecology | 17 (3,68%) | 0 (0%) | 17 (100%) | 25 (1,97%) | 68,00% |
| Haematology | 15 (3,25%) | 10 (66,7%) | 5 (33,3%) | 16 (1,26%) | 93,75% |
| Infectious and tropical disease | 2 (0,43%) | 1 (50%) | 1 (50%) | 6 (0,47%) | 33,33% |
| Emergency Medicine | 5 (1,08%) | 2 (40%) | 3 (60%) | 48 (3,78%) | 10,42% |
| Occupational Medicine and Health | 9 (1,95%) | 4 (44,4%) | 5 (55,6%) | 27 (2,13%) | 33,33% |
| Intensive care medicine | 4 (0,87%) | 3 (75%) | 1 (25%) | 12 (0,94%) | 33,33% |
| Internal Medicine and Immunology | 10 (2,16%) | 5 (50%) | 5 (50%) | 26 (2,05%) | 38,46% |
| Physical Medicine and Rehabilitation | 6 (1,30%) | 0 (0%) | 6 (100%) | 29 (2,28%) | 20,69% |
| Vascular Medicine | 2 (0,43%) | 0 (0%) | 2 (100%) | 4 (0,31%) | 100,00% |
| Nephrology | 10 (2,16%) | 5 (50%) | 5 (50%) | 22 (1,73%) | 45,45% |
| Neurology | 9 (1,95%) | 5 (55,6%) | 4 (44,4%) | 29 (2,28%) | 31,03% |
| Oncology | 21 (4,55%) | 6 (28,6%) | 15 (71,4%) | 44 (3,46%) | 47,73% |
| Pediatrics | 28 (6,06%) | 6 (21,4%) | 22 (78,6%) | 88 (6,93%) | 31,82% |
| Pneumology | 12 (2,60%) | 2 (16,7%) | 10 (83,3%) | 27 (2,13%) | 44,44% |
| Psychiatry | 28 (6,06%) | 5 (17,9%) | 23 (82,1%) | 167 (13,15%) | 16,77% |
| Radiodiagnostics and Imaging | 25 (5,41%) | 15 (60%) | 10 (40%) | 96 (7,56%) | 26,04% |
| Rheumatology | 10 (2,16%) | 2 (20%) | 8 (80%) | 19 (1,50%) | 52,63% |
| Surgical Specialties | | | | | |
| Child Surgery | 1 (0,22%) | 0 (0%) | 1 (100%) | 9 (0,71%) | 11,11% |
| Maxillofacial Surgery | 2 (0,43%) | 2 (100%) | 0 (0%) | 11 (0,87%) | 18,18% |
| Orthopaedic and Trauma Surgery | 15 (3,25%) | 11 (73,3%) | (4 (26,7%) | 43 (3,39%) | 34,88% |
| Urological Surgery | 9 (1,95%) | 9 (100%) | 0 (0%) | 24 (1,89%) | 37,50% |
| Vascular Surgery | 5 (1,08%) | 4 (80%) | 1 (20%) | 15 (1,18%) | 33,33% |
| Visceral and digestive surgery | 12 (2,60%) | 2 (16,7%) | 10 (83,3%) | 37 (2,91%) | 32,43% |
| ENT and cervicofacial surgery | 3 (0,65%) | 3 (100%) | 0 (0%) | 29 (2,28%) | 10,34% |
| Gynecology-Obstetrics | | | | | |
| Gynecology-Obstetrics | 67 (14,5%) | 10 (14,9%) | 57 (85,1%) | 71 (5,59%) | 94,37% |
| Anesthesia & resuscitation | | | | | |
| Anesthesia & resuscitation | 55 (11,90%) | 34 (61,8%) | 21 (38,2%) | 160 (12,60%) | 34,38% |
