# Supplementary material for: Career aspirations among specialty residents in France: a cross-sectional gender-based comparison
Source: BMC Med Educ. 2021 Jan 19;21:63. doi: 10.1186/s12909-021-02494-1 (PMC7816478; doi:10.1186/s12909-021-02494-1)
Supplement: Supplementary file 3 — Additional file 3. Table S2 : Results for all specialties. [file 12909_2021_2494_MOESM3_ESM.docx]

| Table S2 - all specialties | | | | |
| --- | --- | --- | --- | --- |
| *Social and demographic data, and career aspirations, analysis by gender* | | | | |
|  | total n=462 | men n= 173 (37,4%) | women n= 289 (62,6%) | p value |
| Age Mean (DS) | 27,08 (0,09) | 27,06 (1,98) | 27,10 (1,95) | .860 |
| semester of residency (1-10) Mean (DS) | 5,24 (0,12) | 5,18 (2,65) | 5,28 (2,65) | .719 |
| specialty |  |  |  | <.001 |
| medical surgical anesthesia & resuscitation gynaecology-obstetrics pediatrics | 265 (57,4%) 47 (10,2%) 55 (11,9%) 67 (14,5%) 28 (6,1%) | 92 (53,2%) 31 (17,9%) 34 (19,7%) 10 (5,8%) 6 (3,5%) | 173 (59,9%) 16 (5,5%) 21 (7,3%) 57 (19,7%) 22 (7,6%) |  |
| marital status |  |  |  | .626 |
| single attached married | 134 (29,0%) 272 (58,9%) 56 (12,1%) | 53 (30,6%) 97 (56,1%) 23 (13,3%) | 81 (28,0%) 175 (60,6%) 33 (11,4%) |  |
| number of children |  |  |  | .082 |
| 0 1 2 3 >3 | 425 (92,0%) 24 (5,2%) 9 (1,9%) 1 (0,2%) 3 (0,6%) | 160 (92,5%) 9 (5,2%) 1 (0,6%) 0 (0%) 3 (1,7%) | 265 (91,7%) 15 (5,2%) 8 (2,8%) 1 (0,3%) 0 (0,0%) |  |
| academic partner* |  |  |  |  |
| no doesn't know yes | 264/328 (80,5%) 15/328 (4,6%) 49/328 (14,9%) | 102 (85,0%) 5 (4,2%) 13 (10,8%) | 162 (77,9%) 10 (4,8%) 36 (17,3%) | .262 |
| partner support * |  |  |  |  |
| no doesn't know yes | 27/328 (8,2%) 17/328 (5,2%) 284/328 (86,6%) | 13 (10,8%) 7 (5,8%) 100 (83,3%) | 14 (6,7%) 10 (4,8%) 184 (88,5%) | .379 |
| academic father | 62 (13,4%) | 29 (16,8%) | 33 (11,4%) | .069 |
| academic mother | 80 (17,3%) | 27 (15,6%) | 53 (18,3%) | 0,268 |
| *Career aspirations, analysis by gender* | | | | |
|  | total n=462 | men n= 173 (37,4%) | women n= 289 (62,6%) | p value |
| Master of science |  |  |  | .166 |
| unintended don't know wished but not expected desired and planned already achieved | 267 (57,8%) 36 (7,8%) 41 (8,9%) 62 (13,4%) 56 (12,1%) | 90 (52,0%) 12 (6,9%) 20 (11,6%) 29 (16,8%) 22 (12,7%) | 177 (61,2%) 24 (8,3%) 21 (7,3%) 33 (11,4%) 34 (11,8%) |  |
| PhD in science |  |  |  | .120 |
| unintended don't know wished but not expected desired and planned already achieved | 326 (79,6%) 69 (14,9%) 16 (3,5%) 48 (10,4%) 3 (0,6%) | 110 (63,6%) 30 (17,3%) 8 (4,6%) 24 (13,9%) 1 (0,6%) | 216 (74,7%) 39 (13,5%) 8 (2,8%) 24 (8,3%) 2 (0,7%) |  |
| Publication article |  |  |  | .052 |
| none in progress 1 > 1 | 257 (55,6%) 132 (28,6%) 47 (10,2%) 26 (5,6%) | 85 (49,1%) 56 (32,4%) 24 (13,9%) 8 (4,6%) | 172 (59,5%) 76 (26,3%) 23 (8,0%) 18 (6,2%) |  |
| Post-residency |  |  |  | <.001 |
| PHC-attach assistant doesn't know fellowship | 24 (5,2%) 199 (43,1%) 79 (17,1%) 160 (34,6%) | 10 (5,8%) 57 (32,9%) 24 (13,9%) 82 (47,4%) | 14 (4,8%) 142 (49,1%) 55 (19,0%) 78 (27,0%) |  |
| Wants to do research | 117 (25,3%) | 61 (35,3%) | 56 (19,4%) | .001 |
| Wants to teach | 200 (43,3%) | 106 (61,3%) | 94 (32,5%) | <.001 |
| Career envisioned |  |  |  | <.001 |
| private practice hospital staff physician academic medicien doesn't know | 77 (16,7%) 220 (47,6%) 54 (11,7%) 111 (24,0%) | 33 (19,1%) 74 (42,8%) 37 (21,4%) 29 (16,8%) | 44 (15,2%) 146 (50,5%) 17 (5,9%) 82 (28,4%) |  |
| *Priorities, analysis by gender - mean (DS) Question asked: ‘For the following elements, please rank from 1 (least important) to 5 (most important) their importance for your future self- fulfilment?’* | | | |  |
|  | total n=462 | men  n= 173 (37,4%) | women n= 289 (62,6%) | p value |
| Family Life | 4,50 (0,04) | 4,34 (0,89) | 4,60 (0,73) | .001 |
| Leisure | 4,06 (0,04) | 4,06 (0,93) | 4,07 (0,91) | .981 |
| Freedom of timetables | 3,76 (0,05) | 3,50 (1,09) | 3,92 (0,92) | .001 |
| Financial Compensation | 3,63 (0,04) | 3,71 (1,06) | 3,58 (0,88) | .169 |
| Social Recognition | 3,03 (0,05) | 3,06 (1,19) | 3,02 (1,06) | .728 |
| Interest of the professional activity | 4,09 (0,04) | 4,16 (0,76) | 4,05 (0,73) | .134 |
| Intellectual stimulation | 4,18 (0,04) | 4,26 (0,72) | 4,12 (0,77) | .062 |
| Transmission of knowledge | 3,39 (0,05) | 3,55 (1,13) | 3,29 (1,00) | .012 |
| *Potential obstacles to an academic career, in the total population and among those with academic aspirations, analysis by gender* | | | | |
|  | total n=462 | homme  n= 173 (37,4%) | femme  n= 289 (62,6%) | p value |
| Do you get advice from an academic physician about your future career? Yes (n,%) | 212 (45,9%) | 91 (52,6%) | 121 (41,9%) | .016 |
| Do you feel you are supported when you work on a research project? Yes * (n,%) | 145/229 (63,3%) | 60 (34,7%) | 85 (29,4%) | .479 |
| Do you think that it's possible to reconcile (in terms of time) research activity, teaching and clinical practice? Yes (n,%) | 213 (46,1%) | 87 (50,3%) | 126 (43,6%) | .097 |
| Among the academic doctors on staff who you know, would you say that some are models for you? Yes (n,%) | 297 (64,3%) | 119 (68,8%) | 178 (61,6%) | .133 |
| If yes, are they mainly the same sex as you? Yes (n,%) | 150/297 (50,5%) | 83 (69,7%) | 67 (37,6%) | <.001 |
| Have you already experienced discrimination or prejudice due to your gender? Yes (n,%) | 175 (37,9%) | 13 (7,5%) | 162 (56,1%) | <.001 |
| Do you have doubts about your ability to pursue or succeed at an academic career? Yes (n,%) | 239 (51,7%) | 61 (35,3%) | 178 (61,6%) | <.001 |
| Do you think that your career plans would have been different if you were a member of the opposite sex? Yes (n,%) | 134 (29,0%) | 34 (19,7%) | 100 (34,6%) | <.001 |
